# Successful wound treatment using negative pressure wound therapy without primary closure in a patient undergoing highly contaminated abdominal surgery

**DOI:** 10.1186/s40792-018-0493-5

**Published:** 2018-08-01

**Authors:** Takahiro Yoshioka, Yoshitaka Kondo, Toshiyoshi Fujiwara

**Affiliations:** Department of Gastroenterological Surgery, Okayama University Graduate School of Medicine, Dentistry and Pharmaceutical Sciences, 2-5-1 Shikata-cho, Kita-ku, Okayama, 700-8558 Japan

**Keywords:** Contaminated surgery, Crohn’s disease, JANIS, NPWT, Prophylactic NPWT, SSI

## Abstract

**Background:**

The indications for negative pressure wound therapy (NPWT) continue to expand, and NPWT has become a powerful tool for the treatment of interactive wounds. Recently, the use of NPWT over closed incisions has been shown to prevent surgical site infection (SSI) in patients undergoing contaminated or acute care surgery as prophylactic NPWT. In this article, we present our successful experience using NPWT without primary skin closure for wound treatment after a highly contaminated enterological surgery. The procedure we present in this case report is considerably different from the conventional prophylactic NPWT and a novel method in the field of gastrointestinal surgery.

**Case presentation:**

A 33-year-old man with Crohn’s disease underwent a dirty, infected enterological surgical procedure for the treatment of abdominal wall abscess and multiple fistulas around his colonic stoma. The stoma reconstruction and wound debridement resulted in a broad skin defect, and the incision was strategically left open. In addition to the infected wound condition (class IV), Crohn’s disease itself is a risk factor for SSI; consequently, we induced NPWT immediately after the surgery and closed the incision from both ends in a stepwise manner using sutures each time we changed the dressing. This procedure was effective, enabling complete healing and closure at the surgical site on postoperative day 14 without infection or a skin defect.

**Conclusion:**

For highly contaminated enterological surgery, purposely leaving the incision open and starting NPWT immediately after the procedure is an effective strategy for early wound closure and the prevention of SSI.

## Background

Negative pressure wound therapy (NPWT) has enabled dramatic advances in the treatment of incurable wounds during the past two decades [1]. Because surgical site infection (SSI) after abdominal surgery increases the treatment duration and impairs the function of the abdominal wall, NPWT plays a pivotal role in enterological surgery. As the indications for NPWT have been expanding, a number of articles have reported the effects and safety of preventive NPWT over closed incisions, a strategy known as prophylactic NPWT [2–10]. On the other hand, surgical incisions can be difficult to close in one step without creating a skin defect in patients undergoing contaminated enterological surgery because of the need for broad abscess debridement.

Patients with Crohn’s disease often require multiple abdominal surgeries during their lifetimes. The prevention of SSI is even more important in young patients with Crohn’s disease not only to reduce the treatment period, but also to maintain the function of the abdominal wall. We often encounter the need for highly contaminated enterological surgery in young patients with Crohn’s disease. In this article, we present our successful experience treating a dirty, infected wound and describe the usefulness of NPWT induced immediately after surgery without primary skin closure.

## Case presentation

A 33-year-old man was diagnosed as having Crohn’s disease 15 years previously and had undergone a left semi-colectomy and colostomy surgery 2 years later because of an anastomotic stenosis. Unfortunately, an abdominal wall abscess caused by multiple colonic fistulas formed around his stoma several years after the surgery. His physician recommended a second surgery, but the patient refused to consent to the procedure for more than 10 years. Finally, the stoma stenosis progressed, and the patient’s condition became uncontrollable. At that time, the patient agreed to be transferred to our hospital and underwent an operation.

**Fig. 1:**
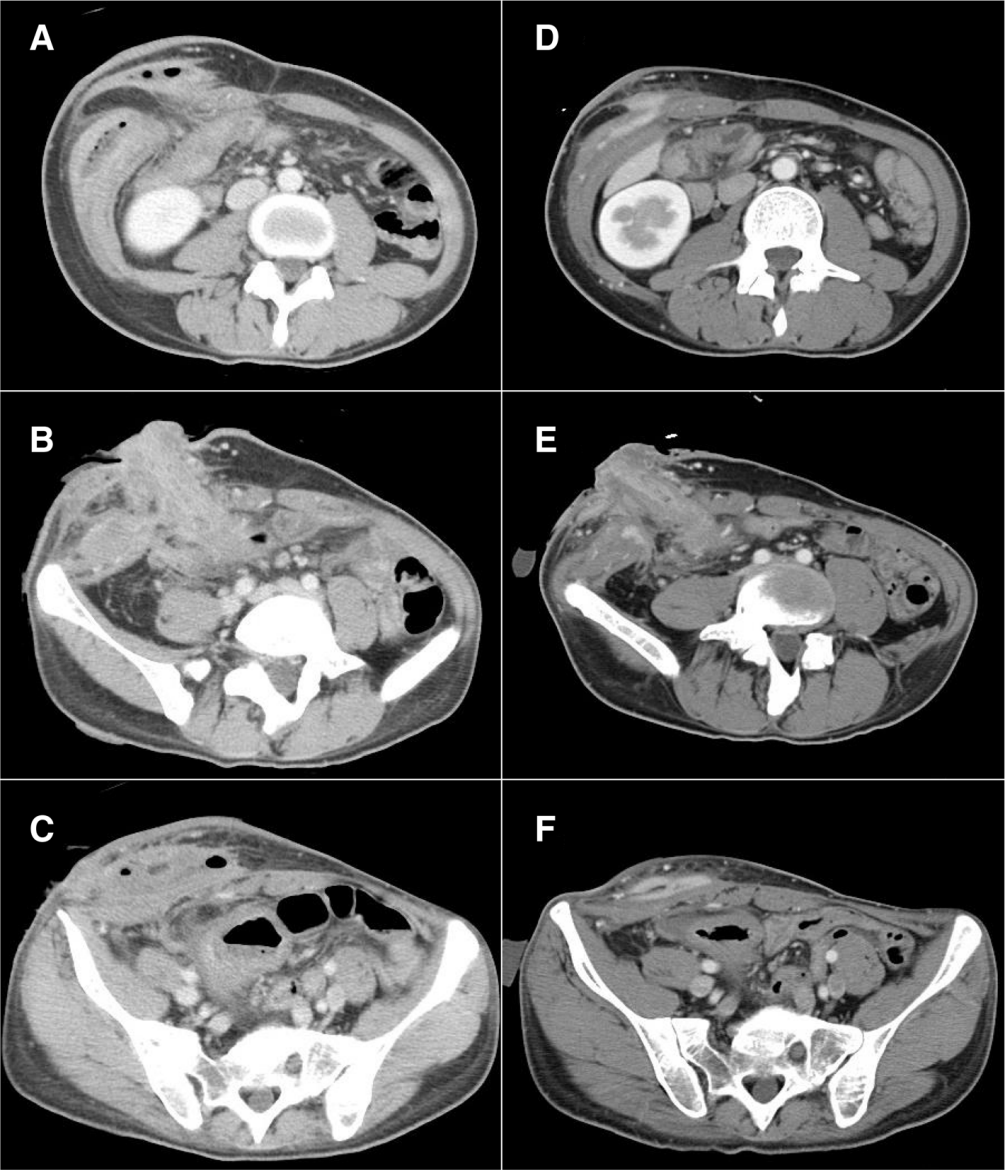
Representative abdominal contrast-enhanced CT scans. **a**–**c** Images obtained 3 weeks before surgery. Abscess cavities have formed in the abdominal wall and inflammatory changes are visible around the stoma site. **d**–**f** Images of CT scans obtained 1 week before surgery. The inflammatory changes have improved considerably, but the abscess cavities remain

Abdominal computed tomography (CT) scans showed the formation of a massive abscess in the rectus abdominis and outer oblique muscle around his ascending colonic stoma and edematous changes in the intraperitoneal and abdominal wall fat tissues (Fig. 1a–c). *Methicillin-resistant Staphylococcus aureus* (MRSA) was detected in a fecal culture examination. However CT scans showed signs of progressive inflammation, we decided to perform conservative treatment before the planned surgical treatment to avoid excessive destruction of the abdominal wall because of the presence of Crohn’s disease. After 2 weeks of conservative treatment with fasting, parenteral nutrition, and antibiotic therapy, a CT scan indicated a considerable improvement in the inflammation around the stoma, though the abdominal wall abscess had persisted (Fig. 1d–f). In addition, almost all the laboratory findings were within the normal ranges including the C-reactive protein (CRP) level, with the exception of anemia (hemoglobin, 9.9 g/dL) and malnutrition (albumin, 3.0 g/dL). Based on these results, we expected to be able to perform the surgery safely.

Colostomy closure, ileocecal resection, abdominal wall abscess and fistula debridement, and ileostomy construction were performed through a 15-cm-long midline incision. The patient’s general condition was classified as class 2 according to the American Society of Anesthesiologists (ASA) physical classification, and the wound was stratified as class IV according to the Centers for Disease Control and Prevention (CDC) classification. The ascending colonic stoma with multiple fistulas was firmly adhered to the surrounding tissue, and the operation required 4 h and 16 min to complete. In addition to the dirty, infected condition, the long operation time also increased the risk of SSI. Furthermore, the skin defect after the resection of the ascending colonic stoma was 10 cm long × 5 cm wide. We were able to close the rectus sheath and the abdominal oblique muscle using an antiviral absorbable suture, but closure of the edematous body surface was challenging. Accordingly, we left the body surface open and covered the wound with wet gauze.

**Fig. 2:**
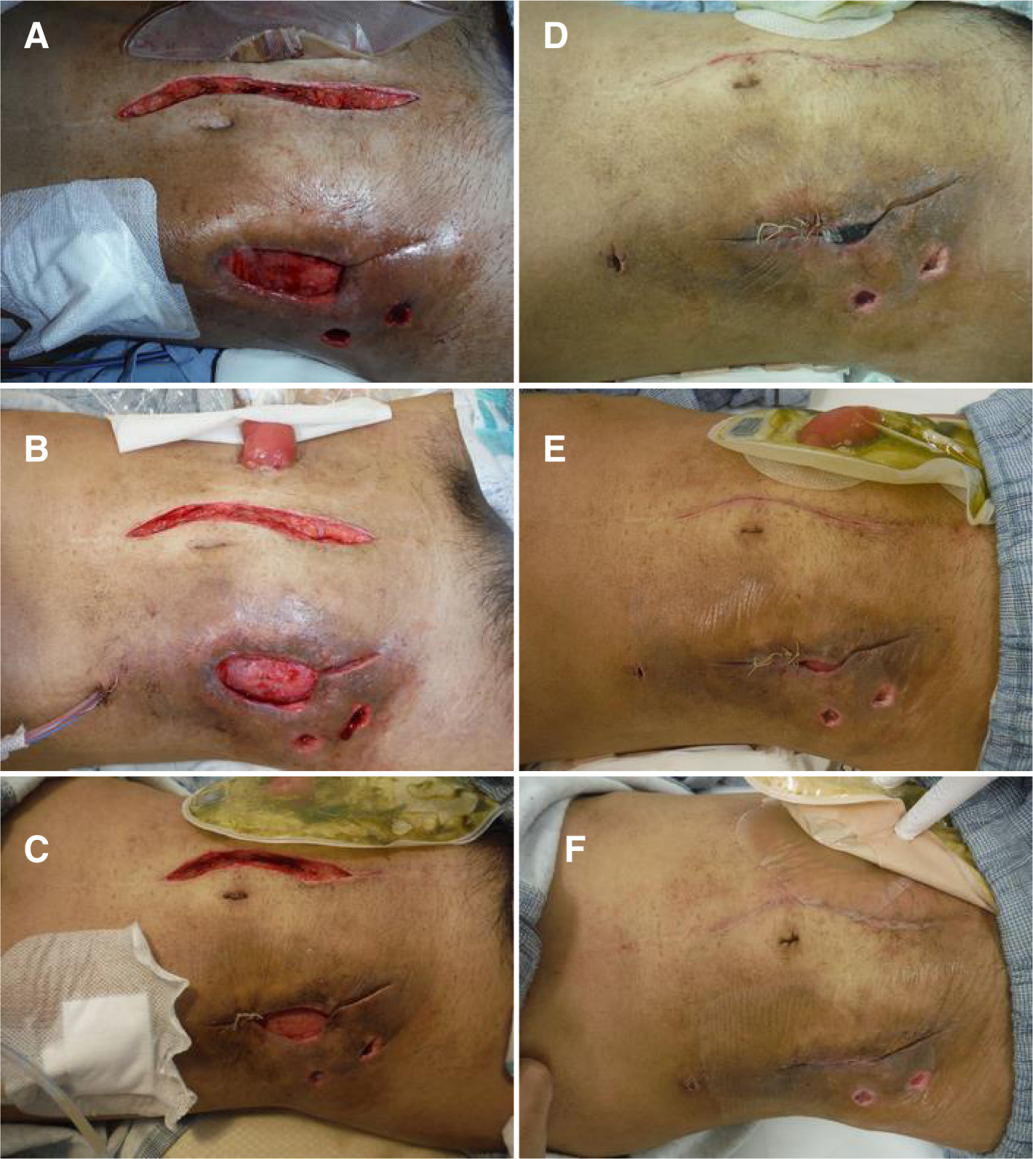
Photographs of the wound. **a** NPWT was induced immediately after surgery. **b** POD 2. **c** POD 4. **d** POD 6. **e** POD 8. **f** The wound had closed completely on POD 14

As soon as the patient returned to his room, we removed all the wet gauze and started NPWT (Fig. 2a). In the present case, we performed NPWT using the V.A.C. (vacuum-assisted closure) system (KCI USA, Inc., San Antonio, TX, USA). The dressing was changed once every 2 days, and the skin defect wound was closed using sutures from both edges in a stepwise manner each time the dressing was changed. As prophylactic antibiotics, 1 g of cefmetazole was intravenously administered once every 12 h for 7 days after the surgery. Because the postoperative course was uneventful, the intraperitoneal drainage tube was removed on postoperative day (POD) 5, and bacterial and mycotic cultures of the drained fluid confirmed the absence of infectious organisms. The condition of the wound, including the skin defect area, improved daily (Fig. 2b–e), and the surgical site was successfully closed without any signs of infection on POD 14 (Fig. 2f). The patient was discharged from the hospital on POD16. A pathological examination of the surgical specimen revealed colonic stenosis and chronic inflammation, but no malignant changes were observed.

### Discussion

A number of clinical practice guidelines or articles have been published as part of efforts to reduce the incidence of SSI [11–16]. Nonetheless, the incidence of SSI remains high for surgical wounds classified as class IV. According to the Japanese Nosocomial Infection Surveillance System (JANIS), the incidence of SSI for colorectal surgery with a stoma construction classified as having a risk index (R.I.) of 2 or 3 was as high as 31.2% (816 incidents among 2611 cases) in 2016. The classification of JANIS’s R.I. was based on the surgical wound classification, the ASA physical status classification, and the operation time. Basically, JANIS’s R.I. is comparable to that of the National Nosocomial Infections Surveillance System (NNIS) [17], but the cutoff value for the operation time was uniquely set in reference to JANIS’s database.

In our department, we induced NPWT in the treatment of exudative wound in September 2012 at first time and experienced the dramatic effect. Accumulating a successful experience, we gradually expanded the indications for NPWT. And in November 2013, we applied NPWT from just after the abdominal surgery. Because we could confirm the safety and usefulness of the wound treatment applying negative pressure from just after abdominal surgeries, we decided to induce this method in patients with inflammatory bowel disease.

The conventional treatments, including subcutaneous drain or nylon drainage, can also drain exudate. However, the procedure in the present case achieves the appropriate wound bed preparation with continuous negative pressure and promotes wound healing far more efficiently as compared with conventional methods.

In addition to the basic mechanisms responsible for the action of NPWT, such as macrodeformation, microdeformation, fluid removal, and an increase in angiogenesis, the detailed molecular mechanism has also been elucidated: the expression profiles for cytokines, chemokines, and growth factor induced by NPWT promote wound bed preparation and healing [18–20]. In the present case, NPWT was performed without primary closure, and this method enabled the uniform application of a negative pressure over the entire cross section of the wound. Compared with closed-incision NPWT, this procedure enabled a more efficient promotion of wound bed preparation and might be even more effective than closed-incision NPWT, which is a breakthrough wound care, taking into account the principle of this procedure. Though whether this procedure can be stratified as prophylactic NPWT is controversial, the procedure is expected to be effective not only for patients with Crohn’s disease, but also for all patients suffering from chronic SSI, particularly those with a high risk of SSI or a thick abdominal wall. On the contrary, we consider that we should avoid to apply this method to those who with low risk of SSI, from the viewpoint of cost-effectiveness.

On the other hand, the method we performed in this case requires frequent foam exchanges and stepwise closure of the wound site. After contaminated abdominal surgery, a large amount of exudate generates from the wound site with this method, applying NPWT without primary skin closure, and surgeons need to respond promptly to troubles such as obstruction of the foam tube or bleeding. For the wound site is closed with dressing, once the bacteria proliferate in the wound site, the foam and drainage tube easily becomes obstructed and the condition of the wound site gets worse and this method rather promotes infection. In order to avoid complications, surgeons need to observe the wound site carefully and frequently. However, if appropriate countermeasures such as increasing the frequency of foam replacement are taken, these troubles can basically be overcome. In facilities that newly introduce NPWT, it is necessary to experience success cases with treatment for exudative wound and expand the indication gradually.

The wound margin can achieve a soft and infection-free condition through the appropriate use of NPWT. After the completion of wound bed preparation, skin defects can then be closed from both ends in a stepwise manner, as in the present case. Without NPWT, defect closure would likely be difficult because of tissue stiffness and subsequent infection.

The abdominal wall has numerous important functions as an organ, such as the protection of the intra-abdominal organs; the maintenance of an upright posture; the support of the spine; assistance when coughing, urinating, or defecating; and the creation of a feeling of fullness to trigger the endpoint of hunger. The absence of an intact abdominal wall results in the loss of all these functions [21, 22]. Since patients with Crohn’s disease often undergo multiple abdominal surgeries during their lifetimes, the prevention of SSI is especially important for young patients with Crohn’s disease to maintain the function of the abdominal wall, not only because Crohn’s disease itself is reportedly an independent risk factor for SSI [23]. The usefulness of NPWT for surgeries related to Crohn’s disease has already been reported [24, 25], and the procedure used in the present case might also contribute to the effective prevention of SSI in highly contaminated surgery in general.

## Conclusions

Purposely leaving the wound open without primary closure and inducing NPWT after contaminated surgery is a promising treatment strategy. This method is likely to be especially suitable for contaminated abdominal surgery in young patients with Crohn’s disease.

## Abbreviations

ASA: American Society of Anesthesiologists
CDC: Centers for Disease Control and Prevention
JANIS: Japanese Nosocomial Infection Surveillance System
NNIS: National Nosocomial Infections Surveillance System
NPWT: Negative pressure wound therapy
POD: Post-operative day
